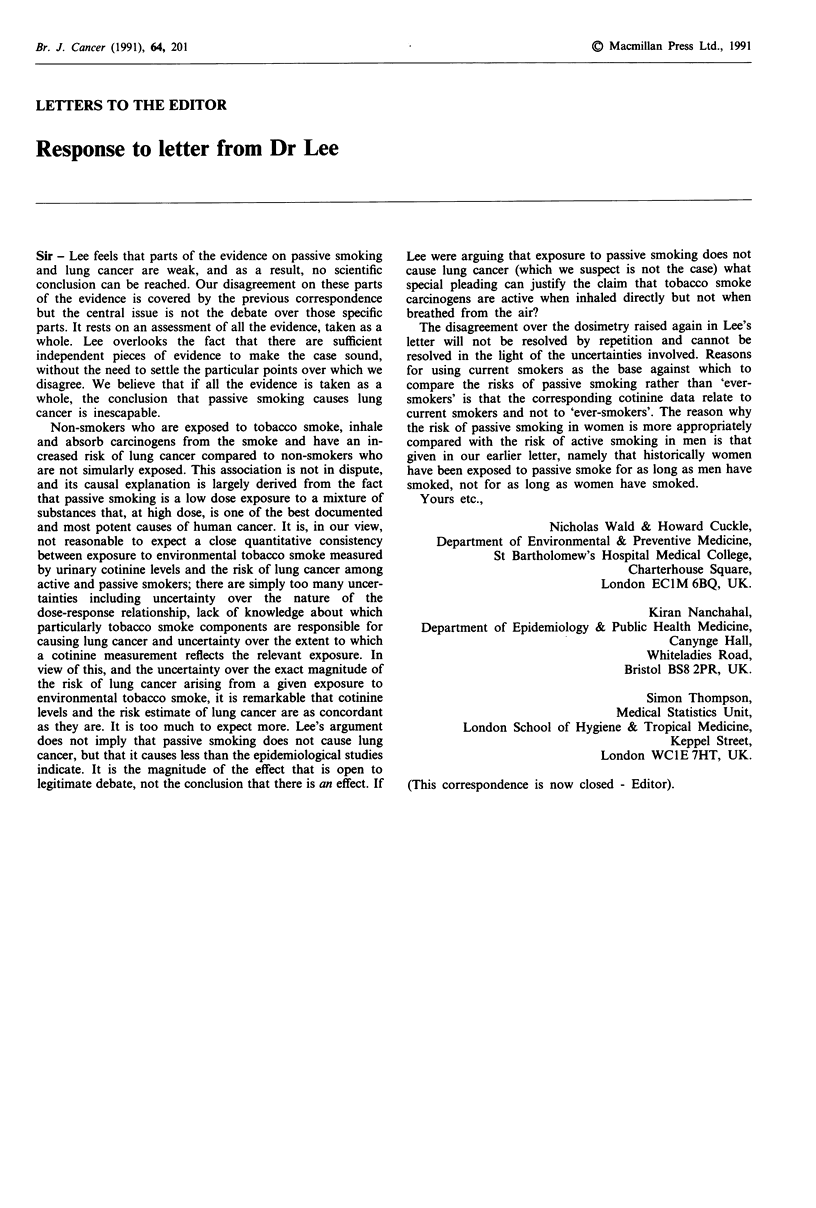# Response to letter from Dr Lee

**Published:** 1991-07

**Authors:** Nicholas Wald, Howard Cuckle, Kiran Nanchahal, Simon Thompson


 © Macmillan Press Ltd., 1991

### LETTERS TO THE EDITOR

### Response to letter from Dr Lee

**Sir** – Lee feels that parts of the evidence on passive smoking and lung cancer are weak, and as a result, no scientific conclusion can be reached. Our disagreement on these parts of the evidence is covered by the previous correspondence but the central issue is not the debate over those specific parts. It rests on an assessment of all the evidence, taken as a whole. Lee overlooks the fact that there are sufficient independent pieces of evidence to make the case sound, without the need to settle the particular points over which we disagree. We believe that if all the evidence is taken as a whole, the conclusion that passive smoking causes lung cancer is inescapable.

Non-smokers who are exposed to tobacco smoke, inhale and absorb carcinogens from the smoke and have an increased risk of lung cancer compared to non-smokers who are not simularly exposed. This association is not in dispute, and its causal explanation is largely derived from the fact that passive smoking is a low dose exposure to a mixture of substances that, at high dose, is one of the best documented and most potent causes of human cancer. It is, in our view, not reasonable to expect a close quantitative consistency between exposure to environmental tobacco smoke measured by urinary cotinine levels and the risk of lung cancer among active and passive smokers; there are simply too many uncertainties including uncertainty over the nature of the dose-response relationship, lack of knowledge about which particularly tobacco smoke components are responsible for causing lung cancer and uncertainty over the extent to which a cotinine measurement reflects the relevant exposure. In view of this, and the uncertainty over the exact magnitude of the risk of lung cancer arising from a given exposure to environmental tobacco smoke, it is remarkable that cotinine levels and the risk estimate of lung cancer are as concordant as they are. It is too much to expect more. Lee's argument does not imply that passive smoking does not cause lung cancer, but that it causes less than the epidemiological studies indicate. It is the magnitude of the effect that is open to legitimate debate, not the conclusion that there is *an* effect. If Lee were arguing that exposure to passive smoking does not cause lung cancer (which we suspect is not the case) what special pleading can justify the claim that tobacco smoke carcinogens are active when inhaled directly but not when breathed from the air?

The disagreement over the dosimetry raised again in Lee's letter will not be resolved by repetition and cannot be resolved in the light of the uncertainties involved. Reasons for using current smokers as the base against which to compare the risks of passive smoking rather than 'ever-smokers' is that the corresponding cotinine data relate to current smokers and not to 'ever-smokers'. The reason why the risk of passive smoking in women is more appropriately compared with the risk of active smoking in men is that given in our earlier letter, namely that historically women have been exposed to passive smoke for as long as men have smoked, not for as long as women have smoked.

Yours etc.,

Nicholas Wald & Howard Cuckle,
Department of Environmental & Preventive Medicine,
St Bartholomew's Hospital Medical College,
Charterhouse Square,
London EC1M 6BQ, UK.

Kiran Nanchahal,
Department of Epidemiology & Public Health Medicine,
Canynge Hall,
Whiteladies Road,
Bristol BS8 2PR, UK.

Simon Thompson,
Medical Statistics Unit,
London School of Hygiene & Tropical Medicine,
Keppel Street,
London WC1E 7HT, UK.

(This correspondence is now closed - Editor).